# Challenges in Implementing Evidence-Based Dementia Care Programs in Community-Based Settings: ADS Plus

**DOI:** 10.1093/geroni/igab046.663

**Published:** 2021-12-17

**Authors:** Joseph Gaugler, Katherine Marx, Lauren Parker, Keith Anderson, Holly Dabelko-Schoeny, Elma Johnson, Elizabeth Albers, Laura Gitlin

**Affiliations:** 1 Johns Hopkins School of Nursing, Baltimore, Maryland, United States; 2 Johns Hopkins Bloomberg School of Public Health, Baltimore, Maryland, United States; 3 University of Texas at Arlington, Arlington, Texas, United States; 4 The Ohio State University, The Ohio State University, Ohio, United States; 5 University of Minnesota School of Public Health, University of Minnesota/ Minneapolis, Minnesota, United States; 6 University of Minnesota, Minneapolis, Minnesota, United States; 7 Drexel University, College of Nursing and Health Professions, Drexel University, Pennsylvania, United States

## Abstract

The Adult Day Service Plus Program (ADS Plus) augments the usual care provided by ADS programs by integrating education, referrals, and problem-solving strategies for family caregivers of persons with dementia. Utilizing a mixed-methods, hybrid effectiveness design, we were in the process of conducting a national evaluation of ADS Plus across xx geographically and culturally diverse programs across the U.S. when the COVID-19 pandemic resulted in the shutdown of almost all of the programs participating in ADS Plus. Qualitative and quantitative data collected during the evaluation suggested that a more robust incorporation of implementation domains and measures (e.g., organizational readiness to change) may have helped avoid some of the challenges related to staff training, fidelity, and other critical intervention delivery aspects. Incorporating implementation science frameworks and measures as early as possible in intervention design may have helped to overcome some of the challenges experienced in ADS Plus.

